# Production of diacetyl by metabolically engineered *Enterobacter cloacae*

**DOI:** 10.1038/srep09033

**Published:** 2015-03-12

**Authors:** Lijie Zhang, Yingxin Zhang, Qiuyuan Liu, Liying Meng, Mandong Hu, Min Lv, Kun Li, Chao Gao, Ping Xu, Cuiqing Ma

**Affiliations:** 1 State Key Laboratory of Microbial Technology, Shandong University, Jinan 250100, People's Republic of China

## Abstract

Diacetyl, a high value product that can be extensively used as a food ingredient, could be produced from the non-enzymatic oxidative decarboxylation of α-acetolactate during 2,3-butanediol fermentation. In this study, the 2,3-butanediol biosynthetic pathway in *Enterobacter cloacae* subsp. *dissolvens* strain SDM, a good candidate for microbial 2,3-butanediol production, was reconstructed for diacetyl production. To enhance the accumulation of the precursor of diacetyl, the α-acetolactate decarboxylase encoding gene (*budA*) was knocked out in strain SDM. Subsequently, the two diacetyl reductases DR-I (*gdh*) and DR-II (*budC*) encoding genes were inactivated in strain SDM individually or in combination to decrease the reduction of diacetyl. Although the engineered strain *E. cloacae* SDM (Δ*budA*Δ*budC*) was found to have a good ability for diacetyl production, more α-acetolactate than diacetyl was produced simultaneously. In order to enhance the nonenzymatic oxidative decarboxylation of α-acetolactate to diacetyl, 20 mM Fe^3+^ was added to the fermentation broth at the optimal time. In the end, by using the metabolically engineered strain *E. cloacae* SDM (Δ*budA*Δ*budC*), diacetyl at a concentration of 1.45 g/L was obtained with a high productivity (0.13 g/(L·h)). The method developed here may be a promising process for biotechnological production of diacetyl.

2,3-Butanediol (2,3-BD) can be efficiently produced by microbial fermentation as a platform and fuel bio-chemical^1^,^2^,^3^,^4^. Many microorganisms such as *Enterobacter*, *Klebsiella*, *Bacillus*, and *Serratia* could be used to produce 2,3-BD from biomass^5^,^6^,^7^,^8^,^9^,^10^. Three key enzymes, including α-acetolactate synthase (ALS), α-acetolactate decarboxylase (ALDC), and 2,3-butanediol dehydrogenase (BDH), are involved in the biosynthesis of 2,3-BD from pyruvate^2^,^4^. Two molecules of pyruvate are condensed to α-acetolactate by ALS. Then, ALDC catalyzes the decarboxylation of α-acetolactate to produce (3*R*)-acetoin ((3*R*)-AC). (3*R*)-AC will be reduced to *meso*-2,3-BD and (2*R*,3*R*)-2,3-BD by *meso*-2,3-BDH and (2*R*,3*R*)-2,3-BDH, respectively^11^. Since *meso*-2,3-BDH and (2*R*,3*R*)-2,3-BDH could also catalyze the reduction of diacetyl to produce AC and 2,3-BD, these enzymes were also called as diacetyl reductase (DR).

α-Acetolactate produced in the 2,3-BD fermentation is unstable and can also be catalyzed through nonenzymatic oxidative decarboxylation to produce diacetyl^12^,^13^. Diacetyl is an important flavor compound responsible for the buttery aroma of many dairy products, and is used as an ingredient in the food industry^14^. Diacetyl could also be produced through chemical methods. However, microbial production of diacetyl is preferred over chemical synthesis as a food or perfume additive due to the safety reasons^15^.

Various microorganisms such as lactic acid bacteria, *Candida glabrata* and *Enterobacter* *aerogenes*, have been used in the production of diacetyl^12^,^16^. For instance, a *Lactococcus lactis* mutant with low ALDC and lactate dehydrogenase activities was able to overproduce diacetyl with a final concentration of 0.52 g/L^17^. Studies by Guo et al. demonstrated that overexpression of NADH oxidase could efficiently tune the lactate and diacetyl production in *L. lactis*^18^. A combination of NADH oxidase overexpression with ALDC inactivation in *L. lactis* could produce diacetyl with a yield of 0.16 mol/mol of glucose, which was the highest yield of diacetyl synthesis till now^14^. Recently, *C. glabrata* CCTCC M202019 was metabolically engineered for diacetyl production. A high titer of 4.7 g/L of diacetyl was achieved with a yield of 0.10 mol/mol and a productivity of 0.07 g/(L·h)^12^. Diacetyl is a byproduct of the 2,3-BD fermentation, but most of the studies mentioned above have been based on strains with low 2,3-butanediol producing capabilities^14^,^19^,^20^. Due to their low efficiency of the glycolytic flux to diacetyl, the productivity and yield of diacetyl using these strains should be further enhanced for industrial production.

In our previous reports, *Enterobacter cloacae* subsp. *dissolvens* SDM can efficiently produce 2,3-BD with a high productivity and a high yield^5^. The key enzymes for 2,3-BD metabolism, including ALS, ALDC, and the two DRs, were annotated based on the genome sequence of the strain^21^. Thus, it might be a good candidate for production of diacetyl through metabolic engineering. In the present work, the ALDC encoding gene *budA* has been knocked out and the DRs encoding genes were also inactivated to construct a diacetyl producer (Figure 1). Fe^3+^ was added to the medium to improve the nonenzymatic oxidative decarboxylation of α-acetolactate to produce diacetyl. Through the metabolic engineering approach described, 1.45 g/L diacetyl was synthesized within 11.3 h with a high yield of 0.21 mol/mol using glucose as substrate.

## Results

### Potential for diacetyl production by *E. cloacae* SDM

2,3-BD exists in three stereoisomeric forms: (2*R*,3*R*)-2,3-BD, (2*S*,3*S*)-2,3-BD, and *meso*-2,3-BD. Recently, the mechanism of 2,3-BD stereoisomer formation was identified in 2,3-BD producing strains including *K. pneumonia*. (2*R*,3*R*)-2,3-BD and *meso*-2,3-BD are mainly produced from the (3*R*)-AC by the reactions catalyzed by (2*R*,3*R*)-2,3-BDH and *meso*-2,3-BDH, respectively^11^. (2*S*,3*S*)-2,3-BD could only be produced by the *meso*-2,3-BDH catalyzed reduction of (3*S*)-AC, which is not an enzymatic decarboxylation product of α-acetolactate but a reduction product of diacetyl^11^. As shown in Figure 2 I-B, the main metabolic products of *E. cloacae* SDM were *meso*-2,3-BD and (2*S*,3*S*)-2,3-BD when glucose was used as the carbon source. Low concentrations of (3*R*)-AC and (3*S*)-AC were also produced under aerobic conditions. (2*S*,3*S*)-2,3-BD could only be produced via the *meso*-2,3-BDH catalyzed two step reduction of diacetyl, the nonenzymatic oxidative decarboxylation product of α-acetolactate. Thus, diacetyl was produced as an intermediate of 2,3-BD biosynthesis in *E. cloacae* SDM. Redirecting more carbon flux toward the 2,3-BD into diacetyl through metabolic engineering of *E. cloacae* SDM might result in an efficient strain for the production of diacetyl.

### Metabolic characteristics of the ALDC mutant of *E. cloacae* SDM

Although there was nonenzymatic oxidative decarboxylation of α-acetolactate in *E. cloacae* SDM, little diacetyl accumulated in the 2,3-BD fermentation process (Figure 2 I-A Figure 2 I-C and Table 1). Since α-acetolactate is mainly subjected to enzymatic conversion to (3*R*)-AC catalyzed by ALDC besides nonenzymatic oxidative conversion to diacetyl, lack of diacetyl accumulation might be due to the high degradation rate of α-acetolactate. Since the enzymatic irreversible reaction drains the available pool of α-acetolactate for diacetyl formation, knockout of the ALDC might be an effective method for the enhancement of diacetyl production in the *E. cloacae* strain SDM.

In this study, *E. cloacae* SDM (Δ*budA*) was constructed by knock-out of the *budA* gene (Gene bank: 13167655) through allele exchange (Figure 2 II-A). The effects of *budA* gene deletion on the ALDC activity and diacetyl formation of strain SDM are shown in Table 1 and Figure 2 II-C, respectively. In the native strain, the ALDC activity towards α-acetolactate was 3.81 ± 0.16 U/mg while little ALDC activity (0.04 ± 0.00 U/mg) was detected in *E. cloacae* SDM (Δ*budA*). After 36 h fermentation, the concentration of diacetyl produced by *E. cloacae* SDM (Δ*budA*) was 59.7 mg/L while only 2.85 mg/L diacetyl was obtained by the native strain SDM (Table 1). Besides diacetyl, (3*R*)-AC, (3*S*)-AC, (2*R*,3*R*)-2,3-BD, (2*S*,3*S*)-2,3-BD, and *meso*-2,3-BD were also detected in the medium (Figure 2 II-B). These results indicate that the diacetyl would also be converted into those compounds in *E. cloacae* SDM (Δ*budA*).

### Inactivation of DR-I in the ALDC mutant of *E. cloacae* SDM

Glycerol dehydrogenase (GDH) belongs to the medium-chain dehydrogenase family and accepts a broad range of substrates. Diacetyl could be reduced to (3*R*)-AC and (2*R*,3*R*)-2,3-BD by the GDH in *K. pneumonia*. A *gdh* gene (Gene bank: 13166340), which exhibits 59% sequence identity with that of *K. pneumonia*, was identified in the genome sequence of *E. cloacae* SDM. In this study, the protein encoded by *gdh* gene was renamed as DR-I due to its diacetyl reduction activity. As shown in Table 1, inactivation of DR-I would result in a lower DR activity of *E. cloacae* SDM (Δ*budA*Δ*gdh*) than that of the strain *E. cloacae* SDM and the mutant strain *E. cloacae* SDM (Δ*budA*). However, the concentration of diacetyl increased modestly to only 326.7 mg/L (Table 1). (3*R*)-AC, (3*S*)-AC, (2*R*,3*R*)-2,3-BD, (2*S*,3*S*)-2,3-BD, and *meso*-2,3-BD would still accumulate during the fermentation (Figure 2 III-B).

### Inactivation of DR-II in the ALDC mutant of *E. cloacae* SDM

The genes that encode ALDC, ALS, and *meso*-2,3-BDH are sequentially clustered in one operon in *E. cloacae* SDM (Figure S1). Our previously studied enzymatic reactions showed that *meso*-2,3-BDH can catalyze the conversion of diacetyl to (3*S*)-AC and further to (2*S*,3*S*)-2,3-BD as well as (3*R*)-AC to *meso*-2,3-BD. In this study, the *meso*-2,3-BDH (renamed as DR-II) encoding gene *budC* (Gene bank: 13167657) was knocked out through the allele exchange in *E. cloacae* SDM (Δ*budA*) (Figure 2 IV-A).

As shown in Table 1, inactivation of DR-II would result in a sharp decrease of DR activity in *E. cloacae* SDM (Δ*budA*Δ*budC*). The concentration of diacetyl increased to 416.1 mg/L after 36 h fermentation (Figure 2 IV-C, Table 1). The *budC* mutant lost the ability to produce (2*S*,3*S*)-2,3-BD and *meso*-2,3-BD (Figure 2 IV-B). This phenotype indicates that the formation of both (2*S*,3*S*)-2,3-BD and *meso*-2,3-BD depends on the activity of DR-II.

Then, DR-I and DR-II were both inactivated in the ALDC mutant of *E. cloacae* SDM (Figure S2). As shown in Table 1, the DR activity would further decrease in the DR-I and DR-II double mutant. However, the glucose consumed, biomass, and concentration of diacetyl would also decrease in the mutant of *E. cloacae* SDM (Δ*budA*Δ*budC*Δ*gdh*). Since the concentration (416.10 mg/L) of diacetyl obtained by *E. cloacae* SDM (Δ*budA*Δ*budC*) was higher than that of other strains, *E. cloacae* SDM (Δ*budA*Δ*budC*) was chosen for further investigation.

### Diacetyl production by *E. cloacae* SDM (Δ*budA*Δ*budC*)

Diacetyl production using *E. cloacae* SDM (Δ*budA*Δ*budC*) was conducted at 37°C in 300-mL shake flasks containing 50 mL medium. The medium was M9 medium supplemented with 18 g/L glucose and 5 g/L yeast extract^22^. The initial pH was 7.4. As shown in Figure 3, 59.8 mg/L diacetyl was obtained from 15 g/L glucose after 12 h of bioconversion. The yield of diacetyl was only at 0.83% of the theoretical value.

The concentration of α-acetolactate produced by *E. cloacae* SDM (Δ*budA*Δ*budC*) was also analyzed during the 12 h of bioconversion. α-Acetolactate of 2.94 g/L was produced. This indicated that the strain *E. cloacae* SDM (Δ*budA*Δ*budC*) showed an almost 32:1 (mol/mol) co-production of α-acetolactate and diacetyl. Thus, diacetyl production could be further enhanced by the transformation of α-acetolactate accumulated in medium.

### Optimization of the addition time of Fe^3+^

In order to achieve higher diacetyl production, non-enzymatic oxidative decarboxylation of α-acetolactate should be enhanced. It was reported that α-acetolactate could be further converted to diacetyl by addition of Fe^3+^^12^,^23^. To study the effect of the addition time of Fe^3+^ on diacetyl production, 20 mM Fe^3+^ was added at 0, 2, 4, 6, 8, 10, and 12 h, respectively. The diacetyl production and glucose consumption were detected after 14 h fermentation.

As shown in Figure 4A and Figure 4B, although addition of Fe^3+^ at 12 h would result in a higher glucose utilization, the highest diacetyl concentration of 1.37 g/L was acquired when 20 mM Fe^3+^ was added at 10 h. Addition of Fe^3+^ at the beginning of the fermentation would inhibit the utilization of glucose and thus decrease the production of diacetyl by *E. cloacae* SDM (Δ*budA*Δ*budC*).

### Batch bioconversion under optimal conditions

Combining the results mentioned above, an optimal system for the production of diacetyl using *E. cloacae* SDM (Δ*budA*Δ*budC*) was developed. Bioconversion was firstly conducted under the conditions mentioned above for 10 h (Figure 5A). Then, 20 mM Fe^3+^ was added in the fermentation medium. As shown in Figure 5B, 1.45 g/L diacetyl was produced in 80 min after the addition of 20 mM Fe^3+^. Glucose of 14.8 g/L was consumed during the bioconversion process. The yield of diacetyl was 0.21 mol/mol glucose. During the two-step bioconversion process, diacetyl was produced with a high productivity of 0.13 g/(L·h).

## Discussion

Diacetyl has a strong buttery flavor and is mainly existed at low concentration in many dairy products, such as butter, beer, and fresh cheeses. Its formation in dairy products mainly results from the catabolism of α-acetolactate during 2,3-BD fermentation by certain species of lactic acid bacteria^14^. Due to the excellent performance of *E. cloacae* SDM as an efficient 2,3-BD producing strain, developing a metabolically engineered strain based on *E. cloacae* SDM through redirecting carbon flux toward the 2,3-BD pathways for the production of diacetyl is quite attractive and promising.

**Figure 1:**
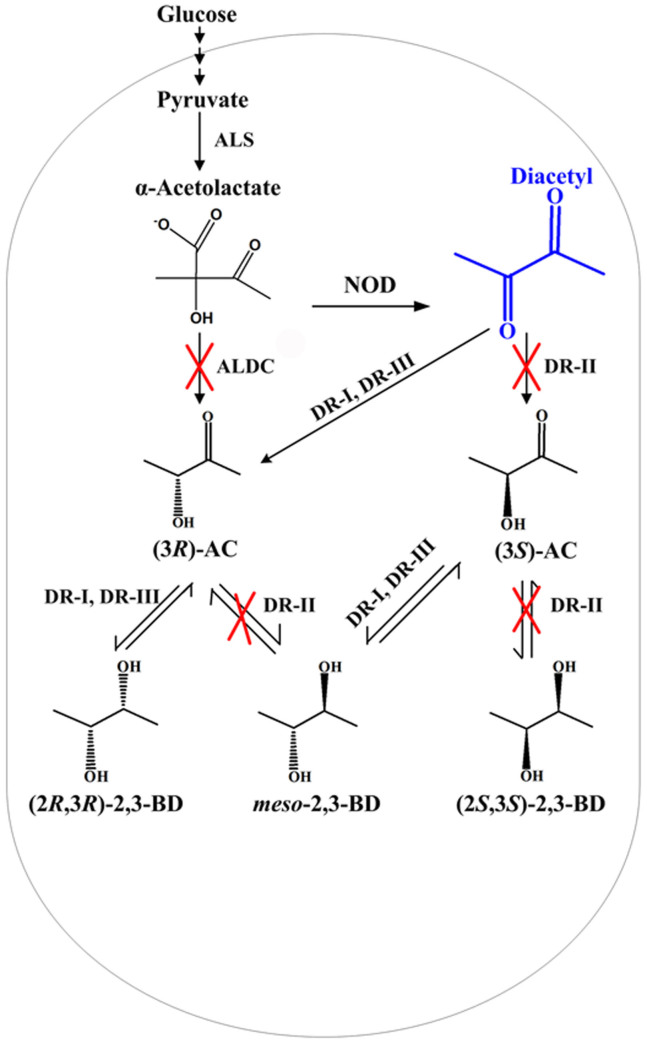
Engineered pathway for diacetyl production in *E. cloacae* SDM. NOD, nonenzymatic oxidative decarboxylation; ALS, α-acetolactate synthase, encoded by *budB*; ALDC, α-acetolactate decarboxylase, encoded by *budA*; DR-I, diacetyl reductase-I (glycerol dehydrogenase), encoded by *gdh*; DR-II, diacetyl reductase-II (*meso*-2,3-butanediol dehydrogenase), encoded by *budC*; DR-III, an undiscovered diacetyl reductase. Crosses represent the enzyme inactivation performed in this study.

In the present study, the diacetyl production from glucose by *E. cloacae* SDM was firstly conducted through two genetic strategies: (i) inactivation of the ALDC gene (*budA*) to avoid enzymatic conversion of the diacetyl precursor α-acetolactate to (3*R*)-AC as described previously^14^ and (ii) inactivation of the DR gene to avoid enzymatic reduction of diacetyl. Two DRs encoding genes (*gdh* and *budC*) were identified in the genome sequence of *E. cloacae* SDM. *E. cloacae* SDM (Δ*budA*Δ*budC*Δ*gdh*) produced diacetyl at a concentration (318.31 mg/L) lower than that of *E. cloacae* SDM (Δ*budA*Δ*budC*) (416.10 mg/L). This result indicates that DR might be important to strain SDM for glucose utilization and cell growth. On the other hand, when DR-I and DR-II were both inactivated in the ALDC mutant, (3*R*)-AC, (3*S*)-AC, and (2*R*,3*R*)-2,3-BD could still be detected (Figure S2), indicating the presence of the third DR (DR-III) responsible for these chemical production in *E. cloacae* strain SDM (Figure 1).

Although 2.94 g/L α-acetolactate was produced from 15 g/L glucose after 12 h of bioconversion, only 59.8 mg/L diacetyl was obtained and the final molar ratio of α-acetolactate and diacetyl was 32:1 (Figure 3), implying an inefficient NOD of α-acetolactate to diacetyl. Thus, besides redirecting carbon flux toward production of α-acetolactate through genetic methods, more efficient chemical conversion of α-acetolactate into diacetyl should also be developed for optimal production of diacetyl. In the study by Gao et al.^12^, an efficient chemical conversion of α-acetolactate to diacetyl could be achieved by addition of Fe^3+^. However, it was indicated that Fe^3+^ would also influence the glucose consumption (Figure 4B) and hence might decrease the diacetyl production during the fermentation process. Thus, the addition time of 20 mM Fe^3+^ was also optimized in the present study. As shown in Figure 4A, when added at 10 h, 20 mM Fe^3+^ could accelerate the NOD of α-acetolactate, and accumulate the highest concentration of diacetyl.

**Figure 2:**
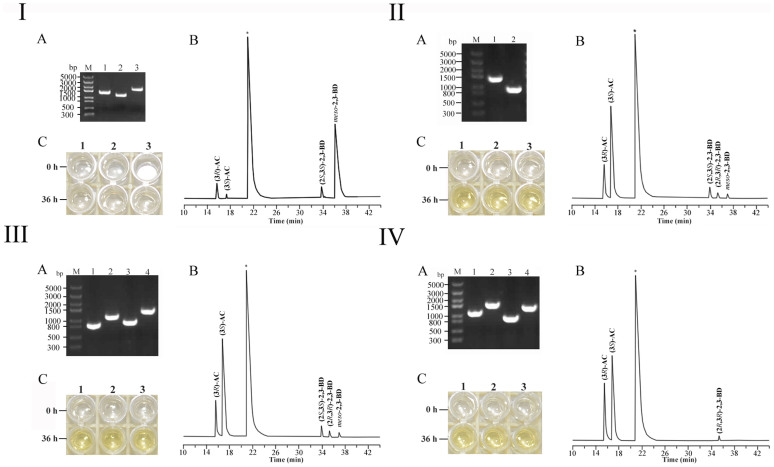
Molecular authentication and metabolic production analysis of *E. cloacae* SDM and its derivatives. Panel I: *E. cloacae* SDM; Panel II: SDM (Δ*budA*); Panel III: SDM (Δ*budA*Δ*gdh*); Panel IV: SDM (Δ*budA*Δ*budC*). (A): Analysis of PCR fragments to confirm disruption of the gene. Lane M, molecular mass standard (Trans5K). (B): Fermentation products identified by GC. (C): Colorimetric detection of diacetyl (1, 2, 3 means all assays were performed by triplicate cultures). I-A: Lane 1–3: *budA*, *gdh*, *budC* products amplified with SDM genomic DNAs as the template. II-A: Lane 1–2, *budA* products amplified with SDM and SDM (Δ*budA*) genomic DNAs as the templates, respectively. III-A: lane 1–2: *gdh* products amplified with SDM (Δ*budA*Δ*gdh*) and SDM genomic DNAs as the templates, respectively; lane 3–4: *budA* products amplified with SDM (Δ*budA*Δ*gdh*) and SDM genomic DNAs as the templates, respectively. IV-A: Lane 1–2: *budC* products amplified with SDM (Δ*budA*Δ*budC*) and SDM genomic DNAs as the templates, respectively; lane 3–4: *budA* products amplified with SDM (Δ*budA*Δ*budC*) and SDM genomic DNAs as the templates, respectively.

**Figure 3:**
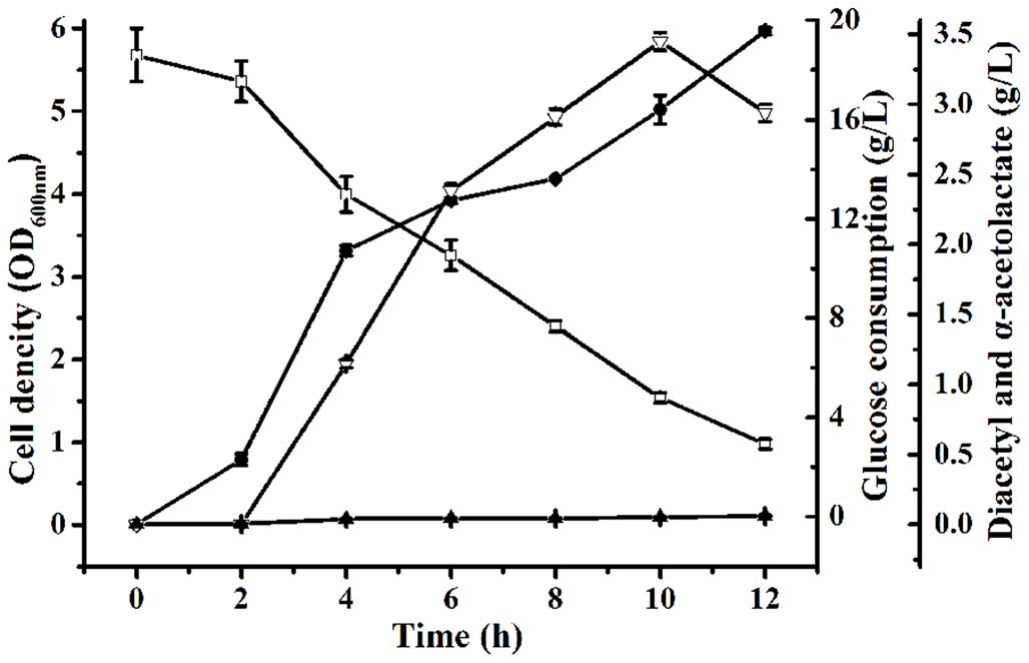
Diacetyl production from glucose using *E. cloacae* SDM (Δ*budA*Δ*budC).* Glucose (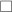), Cell density (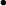), α-Acetolactate (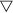), Diacetyl (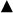). The bioconversion was carried out at 37°C in 300-mL shake flasks containing 50 mL of medium with pH adjusted to 7.4. The initial glucose concentration used was 18 g/L approximately. Error bars indicate standard deviation (n = 3).

**Figure 4:**
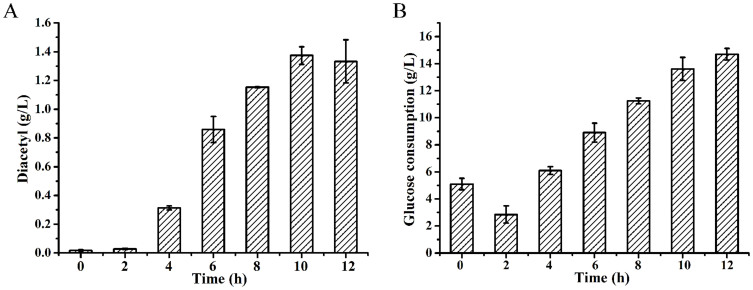
Effects of addition time of Fe^3+^ on diacetyl production (A) and glucose consumption (B). The data were obtained after 14 h fermentation with Fe^3+^ addition at different time points. The bioconversion was carried out at 37°C in 300-mL shake flasks containing 50 mL of medium with pH adjusted to 7.4. The final concentration of Fe^3+^ added to medium was 20 mM. Error bars indicate standard deviation (n = 3).

**Figure 5:**
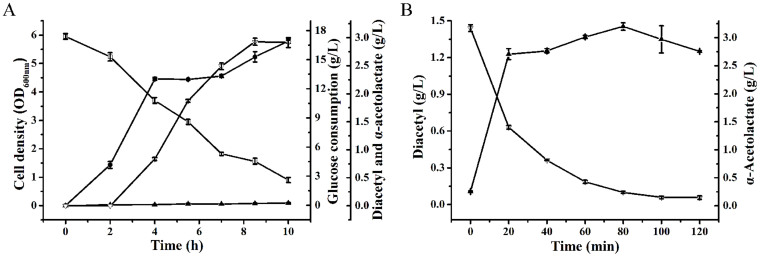
Time course of batch fermentation of diacetyl under the optimization condition. Glucose (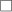), Cell density (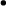), α-Acetolactate (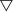), Diacetyl (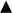). (A) Time course of diacetyl and α-acetolactate production using *E. cloacae* SDM (Δ*budA*Δ*budC*) before Fe^3+^ addition. (B) Time course of diacetyl production after 20 mM Fe^3+^ added at 10 h. The bioconversion was carried out at 37°C in 300-mL shake flasks containing 50 mL of medium with pH adjusted to 7.4. The initial glucose concentration used was 17.8 g/L. Error bars indicate standard deviation (n = 3).

Several biotechnological routes have been used to produce diacetyl (Table 2). Among all of the reported biotechnological processes, the group of Liu obtained the highest diacetyl concentration of 4.7 g/L with a metabolically engineered *C. glabrata*^12^. Efforts have been tried in order to increase the yield of diacetyl through inactivation of ALDC and overexpression of NADH oxidase in *L. lactis*. Using 5 g/L glucose as the substrate, the recombinant *L. lactis* produced 0.38 g/L diacetyl at a high yield of 0.16 mol/mol glucose^14^. In this study, metabolic engineering based on 2,3-BD pathway was used to reconstruct *E. cloacae* SDM as a novel biocatalyst for diacetyl production. Under optimal conditions, the recombinant *E. cloacae* SDM (Δ*budA*Δ*budC*) could produce diacetyl with rather high concentration (1.45 g/L), productivity (0.13 g/(L·h)) and yield (0.21 mol/mol). Both the productivity and yield of diacetyl produced by the recombinant *E. cloacae* were new records for diacetyl production (Table 2). The carbon flux channeled into the diacetyl biosynthetic might be further enhanced since there were still (3*R*)-AC, (3*S*)-AC, and (2*R*,3*R*)-2,3-BD accumulated during the fermentation (Figure 2 IV-C Figure 2 IV-B). This may be accomplished by searching the undiscovered diacetyl reductase, or overexpressing NADH oxidase, which could lead to prevention of NADH dependent reduction of diacetyl.

## Methods

### Chemicals and biochemicals

(2*R*,3*R*)-2,3-BD (98.0%), (2*S*,3*S*)-2,3-BD (99.0%), and *meso*-2,3-BD (98.0%) were purchased from ACROS (The Kingdom of Belgium). Racemic AC, ethyl 2-acetoxy-2-methyl-acetoacetate, and diacetyl were purchased from Sigma. NADH was purchased from Amresco. Restriction enzymes were purchased from TaKaRa Bio Inc. (China). PCR primers were prepared by Sangon (Shanghai, China). FastPfu DNA polymerase and T_4_ DNA ligase were purchased from Transgen Biotech (China) and MBI (USA), respectively. All other chemicals were of analytical grade and commercially available.

### Bacterial strains and plasmids

All the strains and plasmids used in this study are listed in Table 3. *E. coli* DH5α was used for general cloning procedures. The pKR6K was used for gene knock-out in *E. cloacae* strain SDM^24^. *E. coli* S17-1, which is able to host pKR6K and its derivatives, was used for conjugation with *E. cloacae* SDM. Lysogenic broth (LB) medium was used for the culture of *E. coli* and *E. cloacae* SDM. The selection medium in the conjugation experiments was M9 minimal medium supplemented with 1% sodium citrate as the carbon source and 0.05% ammonium chloride as the nitrogen source. Solid LB medium with 10% sucrose was used to select plasmid excision from the chromosome during the gene allelic exchange experiments. Kanamycin was used at a concentration of 50 μg/mL.

### Knock out of the genes in *E. cloacae* SDM

Primers used in this study are listed in Table S1. Isolation of vectors, restriction enzyme digestion, agarose gel electrophoresis, and other DNA manipulations are carried out by standard protocols^25^. Mutants of *E. cloacae* strain SDM were generated by allele exchange using the suicide plasmid pKR6K^24^. The left and right flanking sequences were amplified from *E. cloacae* SDM and then ligated through PCR to get Δ*budA* fragment using primer pairs PΔ*budA*.f (*EcoR*I)/PΔ*budA*.r (overlap), PΔ*budA*.f (overlap)/PΔ*budA*.r (*BamH*I). The gel-purified Δ*budA* fragments were ligated to the pKR6K vector digested with the *EcoR*I and *BamH*I. The resulting plasmid was designated pKΔ*budA*. For conjugation, donor and recipient strains were grown in LB to initial log phase (OD_600 nm_ = 0.5), then collected and mixed at a ratio of 5:1 and spotted on LB plate. After 12 h of conjugation at 37°C, cells were recovered by washing the LB plate with normal saline and plated on the selection medium plates to eliminate the donor strain. The merodiploid (single-crossover) genotype was confirmed by PCR using primers PΔ*budA*.f (*EcoR*I) and PΔ*budA*.r (*BamH*I). Next, a single merodiploid colony was grown overnight in LB medium and appropriate dilutions were plated onto LB agar with 10% (w/v) sucrose, and then incubated overnight at 37°C. Colonies were screened by PCR using primers PΔ*budA*.f (*EcoR*I) and PΔ*budA*.r (*BamH*I). The *budC* and *gdh* mutants of strain SDM were generated by the same way of *E. cloacae* SDM (Δ*budA*).

### Batch fermentation

The batch fermentation was conducted in 300-mL shake flasks containing 50 mL medium. The medium consisted of M9 medium supplemented with 18 g/L glucose and 5 g/L yeast extract. The cultivation was carried out at 37°C and 180 rpm. The initial pH was adjusted to 7.4. Samples were collected periodically to determine the Cell density, concentrations of glucose, diacetyl, and α-acetolactate.

### Enzyme activity assays

For the assays of the activities of ALDC and DR, cells of the strain were grown for 8 h, then centrifuged at 13,000 × g for 5 min, and washed twice with 67 mM phosphate buffer (pH 7.4). Cells were finally resuspended with 67 mM phosphate buffer (pH 7.4) to an OD_600 nm_ of 20, and disrupted with an ultrasonic cell breaking apparatus (Xinzhi, Ningbo, China). Cell debris was removed through centrifugation at 13,000 × g for 15 min. Enzyme activity was assayed in the resulting supernatant.

The activity of ALDC was assayed by detecting the production of AC from α-acetolactate^26^. α-Acetolactate was prepared immediately before use from ethyl 2-acetoxy-2-methyl-acetoacetate according to the protocol supplied by the manufacture. One unit of ALDC activity was defined as the amount of protein that produced 1 μmol of AC per min.

The activity of DR was assayed spectrophotometrically by measuring the change in absorbance at 340 nm corresponding to the oxidation of NADH (ε_340_ = 6,220 M^−1^ cm^−1^) at 30°C using a UV/visible spectrophotometer (Ultrospec 2100 pro, Amersham Biosciences, USA)^27^,^28^. The reaction solution for DR assay contained 5 mM of diacetyl and 0.2 mM of NADH in 67 mM phosphate buffer (pH 7.4). One unit of activity was defined as the amount of enzyme that consumed 1 μmol of NADH per min. The protein concentration was measured by the Lowry method, with bovine serum albumin as the standard^29^.

### Analytical methods

Samples were withdrawn periodically and centrifuged at 12,000 × *g* for 10 min. The Cell density was determined by monitoring the absorbance at 600 nm using a spectrophotometer (LENGGUANG-721, China) after an appropriate dilution. The concentration of glucose was measured enzymatically by a bio-analyzer (SBA-40D, Shandong Academy of Sciences, China) after diluting to an appropriate concentration. The concentrations of 2,3-BD and AC were analyzed by GC as described in Ma et al^6^. The concentrations of α-acetolactate and diacetyl were determined by the methods described in the previous reports^12^,^30^.

## Author Contributions

C.G. and C.M. participated in the design of the study. L.Z., Y.Z., Q.L. and L.M. executed the experimental work. L.Z., M.H., K.L. and M.L. analyzed the data. C.G., C.M. and P.X. contributed reagents and materials. L.Z., C.G., C.M. and P.X. wrote and revised the manuscript. All authors read and approved the final manuscript.

## Supplementary Material

Supplementary Information Supplementary materials--Production of diacetyl using metabolically engineered Enterobacter cloacae

## Figures and Tables

**Table 1:** Enzyme activities, glucose consumption and diacetyl production of *E. cloacae* SDM and its derivatives^a^

Strain	DR (U/mg)	ALDC (U/mg)	Glucose (g/L)	Diacetyl (mg/L)
SDM	14.20 ± 1.11	3.81 ± 0.16	35	2.85 ± 1.57
SDM (Δ*budA*)	5.72 ± 0.23	0.04 ± 0.00	19.5	59.70 ± 11.24
SDM (Δ*budA*Δ*gdh*)	5.29 ± 0.13	0.003 ± 0.00	17	326.66 ± 7.54
SDM (Δ*budA*Δ*budC*)	0.53 ± 0.09	0.004 ± 0.00	17	416.10 ± 13.66
SDM (Δ*budA*Δ*budC*Δ*gdh*)	0.47 ± 0.04	0.003 ± 0.00	14.5	318.31 ± 33.08

DR: diacetyl reductase; ALDC: α-acetolactate decarboxylase.

^a^Data are the means ± standard deviations (SDs) from three parallel experiments.

**Table 2:** Comparison of diacetyl production by different microorganisms

Strain	Engineering strategy	Diacetyl (g/L)	Yield (mol/mol)	Productivity (g/(L·h))	Reference
*L. lactis*	Inactivation of ALDC, overexpression of NADH oxidase	0.38	0.16	0.03	14
*L. lactis*	Random mutagenesis	0.52	–a	0.02	17
*L. rhamnosus*	WT	0.6	0.2	0.06	19
*E. aerogenes*	UV mutation and medium optimization	1.35	0.03	–^α^	16
*L. lactis*	Inactivation of ALDC, overexpression of NADH oxidase	0.36	0.12	0.03	18
*C. glabrata*	Overexpression of ALS, inactivation of ALDC and DR, medium optimization	4.70	0.10	0.07	12
*E. cloacae*	Inactivation of ALDC and DR, Fe^3+^ addition	1.45	0.21	0.13	This study

^a^Not mentioned in the reference.

**Table 3:** Bacterial strain and plasmid used in this study^a^

Strain or plasmid	Characteristic(s)	Source or reference
Strain		
*E. coli* DH5α	F^−^, φ*80* *lacZ*Δ*M15*, Δ(*lacZYA*-*argF*)*U169*, *recA1*, *endA1*, *hsdR17*, *phoA*, *supE44λ^−^*, *thi^−1^*, *gyrA96*, *relA1*	Novagen
*E. coli* S17-1	*recA*, pro, *thi*, conjugative strain able to host λ-pir-dependent plasmids	31
*E. cloacae* SDM	Wild-type	5
SDM (Δ*budA*)	*E. cloacae* SDM *budA* disruption mutant strain	This study
SDM (Δ*budA*Δ*budC*)	*E. cloacae* SDM *budA* and *budC* disruption mutant strain	This study
SDM (Δ*budA*Δ*gdh*)	*E. cloacae* SDM *budA* and *gdh* disruption mutant strain	This study
SDM (Δ*budA*Δ*budC*Δ*gdh*)	*E. cloacae* SDM *budA*, *budC* and *gdh* disruption mutant strain	This study
Plasmid		
pEASYBlunt	Ap^r^, cloning vector	Transgen
pKR6K	Km^r^, gene replacement vector derived from plasmid pK18*mobsacB*, R6K origin, Mob^+^ *sacB*	24
pKΔ*budA*	Km^r^, pKR6K derivative, carries a 587 bp deletion of *budA*	This study
pKΔ*budC*	Km^r^, pKR6K derivative, carries a 639 bp deletion of *budC*	This study
pKΔ*gdh*	Km^r^, pKR6K derivative, carries a 302 bp deletion of *gdh*	This study

^a^Ap^r^, ampicilin resistance; Km^r^, kanamycin resistance.
