# Supplementary material for: Production of diacetyl by metabolically engineered Enterobacter cloacae
Source: Sci Rep. 2015 Mar 12;5:9033. doi: 10.1038/srep09033 (PMC4357014; doi:10.1038/srep09033)
Supplement: Supplementary Information — Supplementary materials--Production of diacetyl using metabolically engineered Enterobacter cloacae [file srep09033-s1.pdf]

Supplementary Materials

Production of diacetyl using metabolically engineered *Enterobacter cloacae*

Lijie Zhang, Yingxin Zhang, Qiuyuan Liu, Liying Meng, Mandong Hu, Min Lv, Kun Li, Chao Gao*, Ping Xu#, and Cuiqing Ma

State Key Laboratory of Microbial Technology, Shandong University, Jinan 250100, People's Republic of China

*Corresponding authors. Mailing address: State Key Laboratory of Microbial Technology, Shandong University, Jinan 250100, People's Republic of China.

Phone/Fax: +86-531-88369463, E-mail: macq@sdu.edu.cn, or jieerbu@sdu.edu.cn

#Present address: School of Life Sciences & Biotechnology, Shanghai Jiao Tong University, Shanghai 200240, People's Republic of China

Table S1 Sequences of primers used in this study[α].

Primer	Sequence (5′—3′)
PΔ*budA*.f (*EcoRI*)	GAATTCGCGAAGACATATTGGCCTC
PΔ*budA*.r (overlap)	TCGTCCAGCACGTCGGTCATGCTCGTCCTCTTCAACTTTAT
PΔ*budA*.f (overlap)	TAAAGTTGAAGAGGACGAGCATGACCGACGTGCTGGACGA
PΔ*budA*.r (*BamHI*)	GGATCCGCTTCCAGTTGACCTACA
PΔ*budC*.f (*SacII*)	CCGAGCTCGTGGTGTCGGTGTCCGGTGA
PΔ*budC*.r (overlap)	GGACATCCCCTGGCCTGAGCCGGTTA
PΔ*budC*.f (overlap)	AGGGGATGTCCGAACCGGAAGATGT
PΔ*budC*.r (*XbaI*)	GGTCTAGAACACCACGAAGGATTTGATG
PΔ*gdh*.f (*BamHI*)	AAAGGATCCATGGACCGTATCATTCAAT
PΔ*gdh*.r (overlap)	CAGATAGGTATTGGCTTCGATGTCGGTGTAAATCACAGAA
PΔ*gdh*.f (overlap)	TTCTGTGATTTACACCGACATCGAAGCCAATACCTATCTG
PΔ*gdh*.r (*EcoRI*)	TTTGAATTCTTACTCCCACTCCTGCAGG

[α]Restriction site used in this study were underlined.

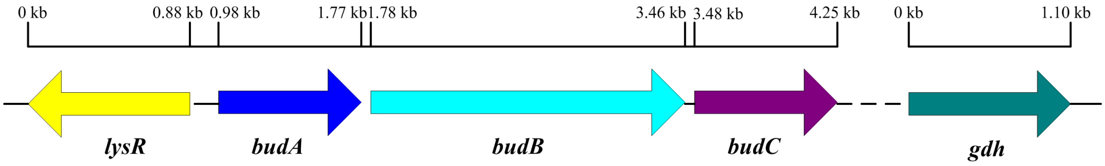

lysR: encoding LysR family transcriptional regulator

budA: encoding α-acetolactate decarboxylase

budB: encoding α-acetolactate synthase

budC: encoding *meso*-2,3-butanediol dehydrogenase

gdh: encoding glycerol dehydrogenase

Figure S1 Location of 2,3-butanediol pathway gene cluster in *E. cloacae* SDM genome.

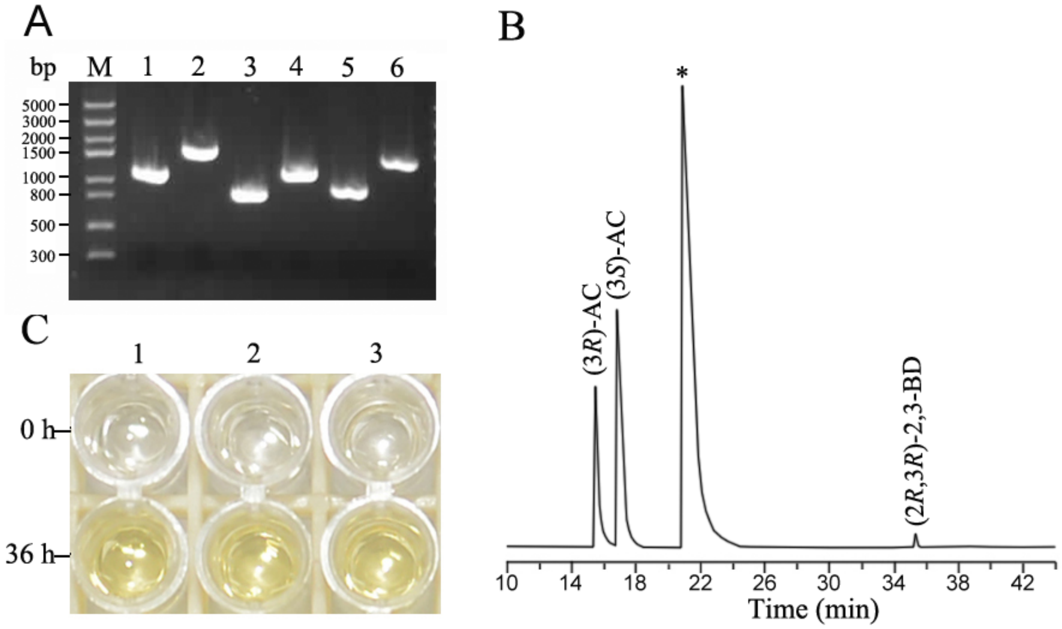

Figure S2 Molecular authentication and metabolic product analysis of *E. cloacae* SDM (Δ*budA*Δ*budC*Δ*gdh*). A: Analysis of PCR fragments to confirm disruption of the gene. Lane M: molecular mass standard (Trans5K); lane 1-2: *budC* products amplified with SDM (Δ*budA*Δ*budC*Δ*gdh*) and *E. cloacae* SDM genomic DNAs as the templates, respectively; lane 3-4: *gdh* products amplified with SDM (Δ*budA*Δ*budC*Δ*gdh*) and *E. cloacae* SDM genomic DNAs as the templates, respectively. lane 5-6: *budA* products amplified with SDM (Δ*budA*Δ*budC*Δ*gdh*) and *E. cloacae* SDM genomic DNAs as the templates, respectively. B: Fermentation products identified by GC. C: Colorimetric detection of diacetyl (1, 2, 3 means all assays were performed by triplicate cultures).
